# Clinicopathological classification of immune checkpoint inhibitor-associated myocarditis: possible refinement by measuring macrophage abundance

**DOI:** 10.1186/s40959-023-00166-1

**Published:** 2023-03-13

**Authors:** Jesus Jimenez, Nicolas Kostelecky, Joshua D. Mitchell, Kathleen W. Zhang, Chieh-Yu Lin, Daniel J. Lenihan, Kory J. Lavine

**Affiliations:** 1 Center for Cardiovascular Research, Department of Medicine, Cardiovascular Division, Washington University School of Medicine, 660 South Euclid Campus, Box 8086, St. Louis, MO 63110 USA; 2 Cardio-Oncology Center of Excellence, Department of Medicine, Cardiovascular Division, Washington University School of Medicine, 660 South Euclid Campus, Box 8086, St. Louis, MO 63110 USA; 3 Department of Pathology and Immunology, Washington University School of Medicine, St. Louis, MO USA; 4 Department of Developmental Biology, Washington University School of Medicine, St. Louis, MO USA

**Keywords:** Cardio-oncology, Immune checkpoint inhibitors, Myocarditis, Macrophage

## Abstract

**Background:**

Immune checkpoint inhibitor (ICI) myocarditis is associated with high morbidity and mortality. While endomyocardial biopsy (EMB) is considered a gold standard for diagnosis, the sensitivity of EMB is not well defined. Additionally, the pathological features that correlate with the clinical diagnosis of ICI-associated myocarditis remain incompletely understood.

**Methods:**

We retrospectively identified and reviewed the clinicopathological features of 26 patients with suspected ICI-associated myocarditis based on institutional major and minor criteria. Seventeen of these patients underwent EMB, and the histopathological features were assessed by routine hematoxylin and eosin (H&E) staining and immunohistochemical (IHC) staining for CD68, a macrophage marker.

**Results:**

Only 2/17 EMBs obtained from patients with suspected ICI myocarditis satisfied the Dallas criteria. Supplemental IHC staining and quantification of CD68^+^ macrophages identified an additional 7 patients with pathological features of myocardial inflammation (> 50 CD68^+^ cells/HPF). Macrophage abundance positively correlated with serum Troponin I (*P* = 0.010) and NT-proBNP (N-terminal pro-brain natriuretic peptide, *P* = 0.047) concentration. Inclusion of CD68 IHC could have potentially changed the certainty of the diagnosis of ICI-associated myocarditis to definite in 6/17 cases.

**Conclusions:**

While the Dallas criteria can identify a subset of ICI-associated myocarditis patients, quantification of macrophage abundance may expand the diagnostic role of EMB. Failure to meet the traditional Dallas Criteria should not exclude the diagnosis of myocarditis.

## Introduction

The revolutionary new class of cancer immunotherapy, known as immune checkpoint inhibitors (ICIs), have resulted in dramatic improvements in outcomes for previously treatment-resistant cancers. A high proportion of cancer patients receive ICIs as either standard clinical practice or through clinical trials that study ICIs as primary therapy or in combination with traditional treatment regimens [1]. Several monoclonal antibodies have been developed to target specific immune checkpoint components. Examples include nivolumab and pembrolizumab that target the programmed cell death protein 1 (PD-1) receptor, atezolizumab that targets programmed cell death ligand 1 (PD-L1), and ipilimumab that targets the cytotoxic T lymphocyte-associated protein 4 (CTLA-4) receptor [2–4]. When activated, these receptors inhibit the intrinsic immune response to cancer cells, allowing cancer cells to avoid detection [5]. By inhibiting PD-1/PD-L1 and CTLA-4 signaling, ICIs permit activation of the cytotoxic immune response against tumor cells.

ICI’s have a wide array of immune-related adverse events including tachyarrhythmias, heart failure, vasculitis, and myocardial infarction [6]. However, fulminant myocarditis is one of the most serious adverse events, with an incidence up to 1% and with fatality rates among the highest of any reported adverse event [7, 8]. The American Society of Clinical Oncology (ASCO) and National Comprehensive Cancer Network (NCCN) have published clinical practice guidelines to aid in diagnosis, including a myocarditis grading system to guide management of patients with ICI myocarditis [9, 10]. However, a definitive diagnosis is often elusive and frequently requires a multi-modal approach [11–13]. From a Cardio-Oncology perspective, considerable effort has been made to standardize the definition of ICI-associated myocarditis by grouping the certainty of the diagnosis into categories of *Possible*, *Probable*, and *Definite* myocarditis [14, 15].

Confirming the diagnosis of ICI myocarditis is particularly challenging given the limited availability of endomyocardial biopsy (EMB) and strict pathological criteria to support this diagnosis. The Dallas criteria for the pathologic diagnosis of myocarditis by EMB requires cardiomyocyte damage in conjunction with the presence of an inflammatory infiltrate that may include lymphocytes, macrophages, eosinophils, neutrophils or a mixture of these immune cells. While these findings can have high specificity for myocarditis, the Dallas criteria have been associated with poor sensitivity in part due to tissue sampling bias and intra-observer variability with interpretation [16]. The diagnostic yield of EMB can be improved with the utilization of immunohistochemistry (IHC) to more readily identify inflammatory infiltrates such as macrophages, which comprise the bulk of tissue resident cardiac immune cells [17, 18]. In healthy hearts, there are roughly 17 macrophages (CD68^+^) per high powered field (HPF), with similar number of macrophages in the early phase following acute myocardial infarction [19, 20]. Previous studies have demonstrated that > 30 CD68^+^ macrophages/HPF are associated with high grade ICI-associated myocarditis [21].

Given the emergence of specific treatments for ICI myocarditis, the ability to make an accurate and expeditious diagnosis represents an important gap in our ability to care for these patients [22–24]. Here, we tested the possibility that quantifying macrophage abundance by IHC may improve the yield of EMB in establishing the diagnosis of ICI-associated myocarditis.

## Methods

### Patient selection

In this single-center retrospective case series, 26 patients with a suspected clinical diagnosis of ICI-associated myocarditis were identified from November 2017 through March 2022. ICI myocarditis was suspected in the setting of symptoms such as dyspnea, palpitations, and chest pain in conjunction with suggestive cardiac biomarkers, ECG, and/or TTE abnormalities not attributable to an alternate non-myocarditis diagnosis [14]. Each case was independently reviewed by two cardiologists and the clinical diagnosis of ICI-associated myocarditis was categorized as: *Definite* (EMB positive OR CMR positive plus 2 minor criteria), *Probable* (CMR positive plus 1 minor criterion OR ≥ 3 minor criteria and EMB/CMR not obtained), or *Possible* (CMR positive without minor criteria OR ≤ 2 minor criteria) [15]. By consensus among institutional experts and integrating ASCO and NCCN guidelines, major criteria for ICI-associated myocarditis included: 1) positive EMB (based on the Dallas Criteria), and 2) positive CMR (by updated Lake Louise criteria [25]). Minor criteria included: 1) ECG changes (significant T wave changes indicating ischemia, any new conduction abnormality or new supraventricular or ventricular tachyarrhythmias), 2) elevated cardiac troponin above institutional normal value, 3) TTE abnormality (wall motion abnormality, left ventricular (LV) dysfunction with EF < 50%, or new/worsening pericardial effusion), and 4) inflammation such as myositis, myasthenia gravis or other major organ inflammation related to recent ICI use.

Demographics, medical comorbidities, cancer treatment history, cardiovascular (CV) risk factors, and medications at the time of first ICI treatment were extracted from the electronic medical record. The CMR report, cardiac related biomarkers, TTEs and ECGs were obtained at the time of suspected myocarditis. TTEs and ECGs were retrieved from digital archive and re-analyzed in accordance with published guidelines [26]. The study was approved by the Washington University in St. Louis institutional review board (ID #201,909,079) and the requirement for written informed consent was waived.

### Histologic staining and evaluation

Among 17 patients who underwent EMB, three to five pieces of endomyocardial tissue were obtained during routine clinical practice for each patient. EMB specimens were examined microscopically via routine H&E staining as well as IHC for CD68 (KP-1 clone, Cell Marque, Rocklin CA). Staining was performed per manufacturer protocol in Clinical Laboratory Improvement Amendments (CLIA)-accredited clinical laboratories, with appropriate positive and negative controls. EMB specimens were retrospectively evaluated by a single pathologist.

H&E sections were evaluated for evidence of myocarditis using the Dallas criteria [16], and a diagnosis of myocarditis was rendered with the presence of cardiomyocyte damage/necrosis with associated inflammatory infiltrates that included a mixture of macrophages and lymphocytes. Cases were denoted as H&E positive if the pathologic sample met the Dallas criteria. Serial sequential IHC sections were evaluated for increased presence of CD68^+^ interstitial macrophages compared to the degree of scattered residential macrophages seen in the healthy heart and in patients with suspected ICI-associated myocarditis. Cases were denoted as IHC positive if there were greater than 50 interstitial CD68-stained macrophages/hpf in hot spot regions (based on 1 standard deviation from 30 macrophages in high grade ICI-associated myocarditis and number of macrophages in healthy and post-injury hearts [19–21]).

The clinical diagnosis of ICI-associated myocarditis was re-assessed by the same two cardiologists using the supplemental IHC analyses, according to the diagnostic algorithm outlined above.

### Statistical analysis

Continuous variables are reported as mean with standard deviation. Categorical variables are reported as total numbers and percentages. For continuous variables, data with 3 groups were compared using analysis of variance (ANOVA) with Bonferroni post-hoc analysis. For categorical variables, data were compared using Fisher’s exact test. For correlation between cell count and biomarkers, Spearman correlation coefficient analysis was performed. For survival curves, data were compared using the Mantel-Cox log-rank test. Values of *P* < 0.05 were considered statistically significant.

## Results

### Histological analysis of ICI-associated myocarditis

**Fig. 1:**
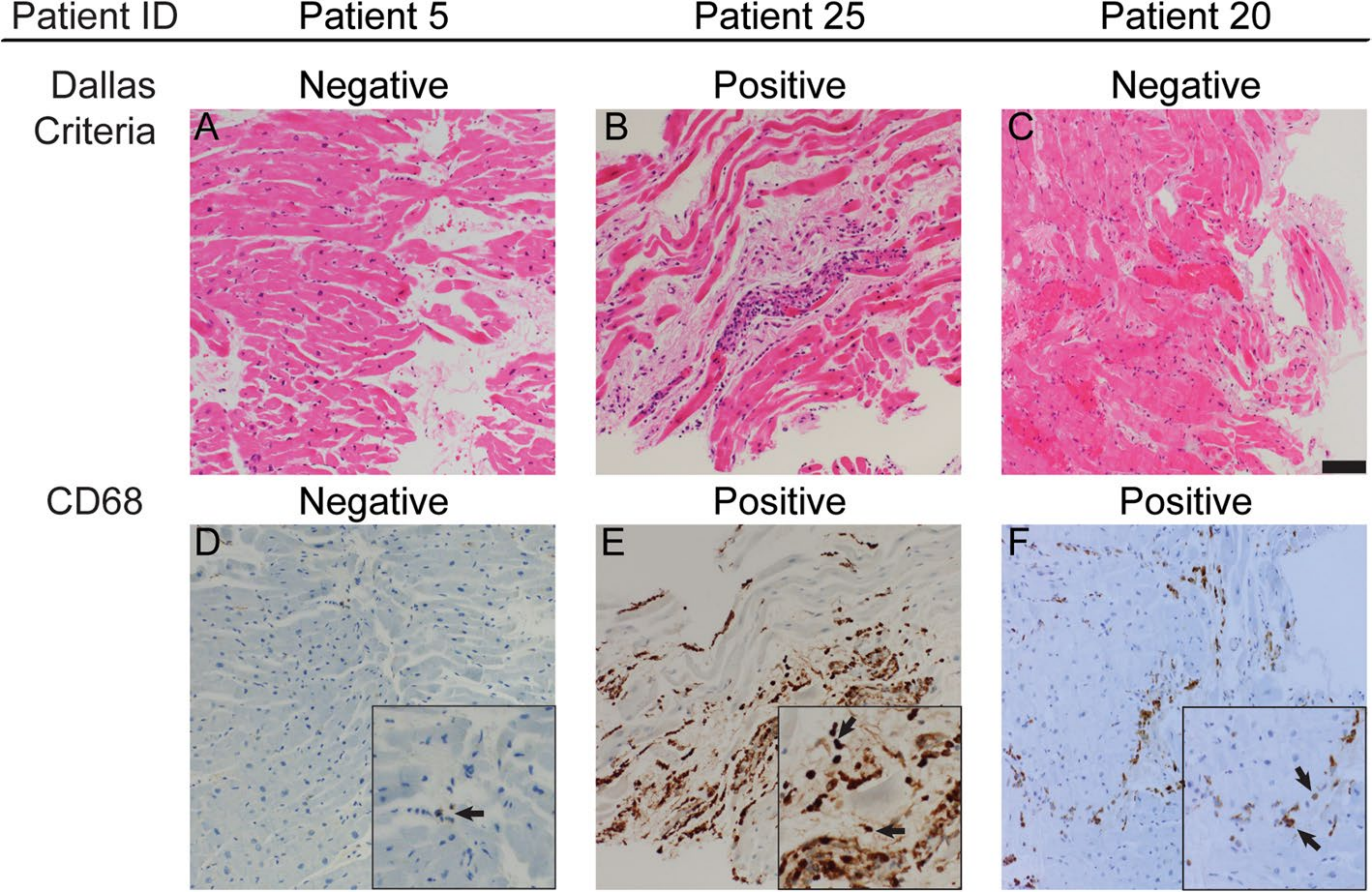
Histology and immunohistochemistry (IHC) of endomyocardial biopsies from patients treated with immune checkpoint inhibitors. Representative areas of each respective biopsy sample that underwent staining from serial sequential sections are shown. The hematoxylin and eosin (H&E)-stained slides did not show definite evidence of myocarditis by Dallas criteria in either **A** Patient 5 or **C** Patient 20 but did demonstrate cardiomyocyte necrosis and loss with increased inflammatory infiltrates in **B** Patient 25. The CD68 stain showed an increased number of interstitial macrophages that overlap with the inflammatory cell infiltration and the cardiomyocyte necrosis perceived in H&E-stain in **E** Patient 25. CD68 macrophage stain also showed an increased number of interstitial macrophages in **F** Patient 20 but not in **D** Patient 5. Insets show magnified views with arrows highlighting representative CD68 stained macrophages. Scale bar = 100 µm. ID = identification

In the 17 patients who underwent EMB, histological examination was performed with at least three levels of H&E-stained slides for assessment using the Dallas criteria. Two patients met the definition of myocarditis using routine H&E stain, having both cardiomyocyte injury/necrosis and focal inflammatory infiltrate comprised of macrophages and lymphocytes (Fig. 1B). Supplemental IHC staining for CD68 in serial sequential sections demonstrated interstitial macrophage infiltration in these two cases (Fig. 1E). In the remaining 15 EMBs that did not meet the Dallas criteria for myocarditis on H&E staining (Fig. 1A, C), supplemental IHC staining for CD68 demonstrated increased macrophage infiltration in 7 samples (Fig. 1F). EMBs demonstrating greater than 50 macrophages/HPF were considered positive by IHC criteria. Eight samples were classified as negative for IHC due to staining showing less than 50 macrophages/HPF (Fig. 1D).

### Clinical myocarditis classification

**Fig. 2:**
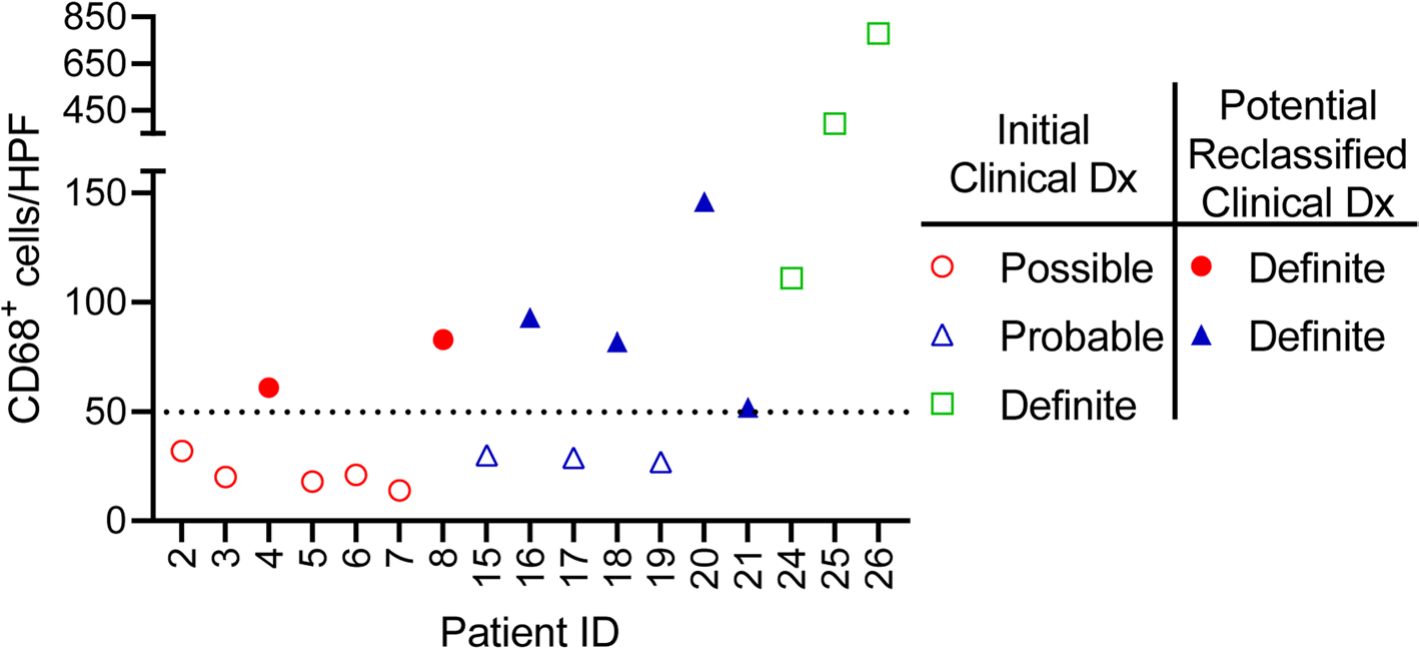
Interstitial macrophage cell counts in endomyocardial biopsies (EMB). Plot of CD68 positive macrophages per high powered field (HPF) from EMBs of patients with clinical diagnosis of *Possible* (red circles), *Probable* (blue triangles), and *Definite* (green squares) myocarditis. The dotted line at 50 macrophages/HPF demarcates the CD68^+^ cell count threshold to consider a sample as immunohistochemistry positive and support reclassification of the initial clinical diagnosis (Dx)

In patients that underwent EMB, the initial clinical diagnosis was based on the major criteria for ICI-associated myocarditis, which requires a positive EMB based on the Dallas Criteria (see Methods). Based on positive CD68 IHC staining, we show potential clinical reclassification of patients from *Possible* or *Probable* to *Definite* myocarditis (Fig. 2). Two of the 8 patients originally classified as *Possible* myocarditis could be reclassified as *Definite*, given pathologic support of the diagnosis through evidence of cardiac inflammation with CD68 staining. Four of the 13 patients originally classified as *Probable* could be reclassified as *Definite* after confirming inflammation with CD68 staining of the EMB. In combination with the 2 patients who met both Dallas criteria and positive CD68 staining, a total of 9 patients could now meet *Definite* criteria through biopsy criteria. Two patients did not undergo biopsy but met *Definite* criteria through clinical criteria and imaging confirmation. Nine patients remained classified as *Probable* myocarditis and 6 remained classified as *Possible* myocarditis before and after CD68 staining. Collectively, the mean age for all patients was 64 ± 12 years and 38% were female (Table 1). Less than 50% of patients had prior coronary artery disease or cardiomyopathy and over 40% of patients were on at least one cardiovascular medication. No significant differences in clinical features were observed across the classifications of ICI-associated myocarditis.

**Table 1:** Patient characteristics – cardiovascular

	***All*** **(*n* = 26)**	***Possible* (*n* = 6)**	***Probable* (*n* = 9)**	***Definite* (*n* = 11)**	***P***	***P (Pos vs Pro)***	***P (Pos vs Def)***	***P (Pro vs Def)***
**Age at clinical diagnosis (yr)**	64 ± 12	65 ± 8	63 ± 9	65 ± 16	0.904			
**Sex**								
Female	10 (38)	1 (17)	4 (44)	5 (45)		0.999	0.333	0.637
Male	16 (62)	5 (83)	5 (56)	6 (55)				
**Race**								
Black or African American	3 (12)	0	2 (22)	1 (9)		0.486	0.999	0.566
White, non-Hispanic	23 (88)	6 (100)	7 (78)	10 (91)				
**Cardiovascular Risk Factors**								
History of smoking	17 (65)	6 (100)	2 (22)	9 (82)		**0.0070**	0.515	**0.0216**
Hypertension	19 (73)	4 (67)	8 (89)	7 (64)		0.525	0.999	0.319
Diabetes mellitus	6 (23)	1 (17)	3 (33)	2 (18)		0.604	0.999	0.617
**Coronary Artery Disease**	12 (46)	3 (50)	2 (22)	7 (64)		0.329	0.645	0.092
**Prior Cardiomyopathy (any)**	8 (31)	3 (50)	1 (11)	4 (36)		0.559	0.999	0.282
**Stroke**	2 (8)	0	0	2 (18)		0.999	0.515	0.479
**Cardiovascular Medications**								
Statin	11 (42)	3 (50)	3 (33)	5 (45)		0.622	0.999	0.670
Aspirin	15 (58)	4 (67)	4 (44)	7 (64)		0.608	0.999	0.653
Beta-blockers	13 (50)	5 (83)	1 (11)	7 (64)		**0.0110**	0.600	**0.0281**
RAAS inhibitors	18 (69)	6 (100)	5 (56)	7 (64)		0.103	0.237	0.999

Values are expressed as mean ± standard deviation or *n* (%). Fisher’s exact test was used when comparing categorical variables and Student’s t-test or analysis of variance when comparing continuous variables. *P* values < 0.05 were considered significant. *Possible*, *Probable*, and *Definite* are based on the proposed reclassified myocarditis patient cohorts

*RAAS *  Renin–angiotensin–aldosterone system, *yr* years

### Cardiac diagnostic testing

**Fig. 3:**
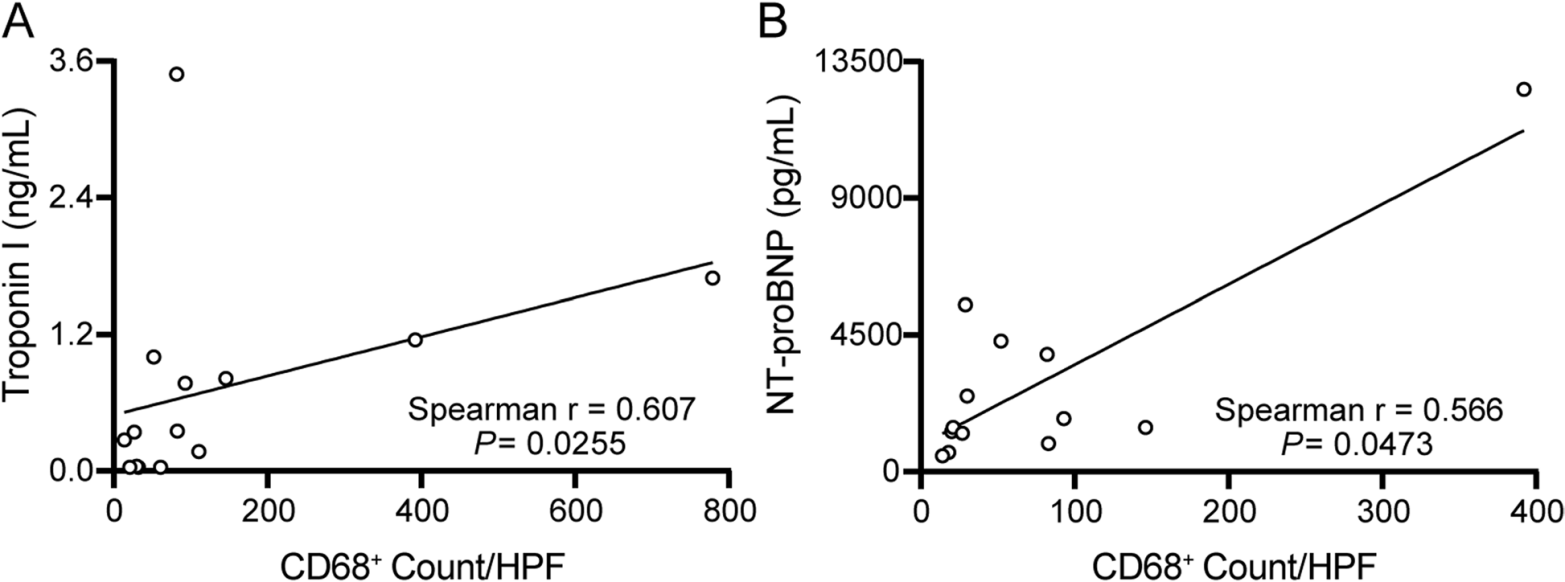
Correlation of cardiac biomarkers and macrophage cell counts. **A** Cardiac troponin I and **B** N-terminal pro-brain natriuretic peptide (NT-pro BNP) levels plotted against corresponding CD68 positive macrophage cells counts per high powered field (HPF). Data were compared using Spearman correlation coefficient analysis with values of *P* < 0.05 being considered statistically significant

Overall, 16 patients underwent CMR and had an average left ventricular ejection fraction (LVEF) of 42%. Eight patients met Lake Louise Criteria for myocarditis (Table 2). Baseline TTE before ICI treatment was available for 20 patients with an average LVEF of 57%. The post ICI treatment TTE had an average LVEF of 43% with a decrease in LVEF of 14% from baseline. Several abnormal findings were seen across all groups, including 1) reduction in LVEF, wall motion abnormalities, or new/worsening pericardial effusions in 18/26 patients (69%), 2) ECG abnormalities such as ischemic T wave changes, new conduction abnormality or new tachyarrhythmias in 20/26 patients (91%), 3) cardiac troponin I elevation in 16/24 patients (67%) and 4) inflammation including myositis or other major organ inflammation in 4/26 patients (15%). NT-proBNP was elevated in all patients at the time of clinical diagnosis and CRP was elevated in 12/18 patients (67%). We identified a significant correlation between CD68^+^ macrophage abundance and serum biomarkers of cardiac injury including troponin I (*P* = 0.026) and NT-proBNP (*P* = 0.047) (Fig. 3A, B).

**Table 2:** Patient characteristics – diagnostic testing

	***All***	***Possible***	***Probable***	***Definite***	***P***	***P (Pos vs Pro)***	***P (Pos vs Def)***	***P (Pro vs Def)***
**Cardiac MRI**								
LVEF (%)^a^	42 ± 20	37 ± 13	47 ± 20	41 ± 22	0.756			
Late gadolinium enhancement^b^	8 (57)	2 (50)	2 (67)	4 (57)		0.999	0.999	0.999
Wall motion abnormality^a^	10 (63)	3 (75)	3 (60)	4 (57)		0.999	0.999	0.999
**Transthoracic echocardiography**								
Pre-ICI LVEF (%)^c^	57 ± 13	44 ± 16	61 ± 7	61 ± 10	**0.0382**	0.066	0.065	0.999
Post-ICI LVEF (%)^d^	43 ± 19	38 ± 14	44 ± 21	45 ± 19	0.778			
Change in LVEF from baseline (%)^e^	-15 ± 17	-4 ± 14	-19 ± 18	-17 ± 16	0.419			
Wall motion score index^f^	24 ± 8	25 ± 9	26 ± 9	23 ± 7	0.575			
Pericardial effusion^g^	7 (29)	2 (33)	3 (43)	2 (18)		0.999	0.517	0.617
**ECG changes (any)**^h^	20 (91)	3 (75)	7 (88)	10 (100)		0.999	0.286	0.444
Heart block	6 (27)	2 (50)	1 (13)	3 (30)		0.236	0.580	0.588
Arrhythmias	12 (55)	1 (25)	4 (50)	7 (70)		0.576	0.245	0.631
ST/T wave changes	13 (59)	1 (25)	6 (75)	6 (60)		0.222	0.559	0.638
**Organ inflammation (any)**^i^	5 (19)	1 (17)	3 (33)	1 (9)		0.604	0.999	0.285
**Peak Biomarkers**								
Troponin I (ng/mL)^j^	4.4 ± 17.4	0.2 ± 0.2	12 ± 29	0.9 ± 0.9	0.357			
NT-proBNP (pg/mL)^k^	9,896 ± 18,841	1,786 ± 969	17,267 ± 24,549	8,791 ± 16,928	0.398			
CRP (mg/L)^l^	79 ± 79	106 ± 74	72 ± 68	76 ± 87	0.842			

Values are expressed as mean ± standard deviation or *n* (%). Fisher’s exact test was used when comparing categorical variables and Student’s t-test or analysis of variance when comparing continuous variables. *P* values < 0.05 were considered significant. The *n* are defined as the following for *All*, *Possible*, *Probable*, and *Definite,* respectively: ^a^*n* = 16, 4, 5, 7; ^b^*n* = 14, 4, 3, 7; ^c^*n* = 20, 5, 7, 8; ^d^*n* = 25, 5, 9, 11; ^e^*n* = 18, 4, 6, 8; ^f^*n* = 17, 2, 7, 8; ^g^*n* = 24, 4, 9, 11; ^h^*n* = 22, 4, 8, 10; ^i^*n* = 26, 6, 9, 11; ^j^*n* = 24, 5, 8, 11; ^k^*n* = 22, 5, 7, 10; ^l^*n* = 18, 3, 7, 8. *Possible*, *Probable*, and *Definite* are based on the proposed reclassified myocarditis patient cohorts. Heart block included non-specific intraventricular block, right bundle branch block, left bundle branch block, or left anterior fascicular block

*CRP* C-reactive protein, *ECG* Electrocardiogram, *ICI* Immune checkpoint inhibitors, *LVEF* Left ventricle ejection fraction, *MRI* Magnetic resonance imaging, *NT-proBNP* N-terminal pro-brain natriuretic peptide

### Cancer characteristics and clinical outcomes

The most common cancer types were non-small cell lung cancer, renal cell carcinoma, and melanoma (Table 3). Patients often receive ICIs following or in conjunction with standard chemotherapy and/or radiation. Fifty-eight percent of patients underwent radiation of which 15% received chest radiation. Eighty-eight percent of patients had prior cancer treatment with 7% receiving anthracyclines and 27% vascular endothelial growth factor (VEGF) inhibitors. The majority of patients were treated with ICI monotherapy that included nivolumab (31%) and pembrolizumab (31%) while 27% were treated with combination therapy comprised of nivolumab and ipilimumab.

**Table 3:** Patient characteristics – cancer

	***All***	***Possible***	***Probable***	***Definite***	***P***	***P (Pos vs Pro)***	***P (Pos vs Def)***	***P (Pro vs Def)***
**Primary Cancer Type**^a^								
Non-small cell lung cancer	5 (19)	1 (17)	1 (11)	3 (27)		0.999	0.999	0.591
Renal cell carcinoma	5 (19)	2 (33)	3 (33)	0		0.999	0.110	0.074
Melanoma	6 (23)	2 (33)	0	4 (36)		0.143	0.999	0.094
Other cancer	10 (38)	1 (17)	5 (56)	4 (36)		0.287	0.600	0.653
**Prior Radiation**^a^								
Chest radiation	4 (15)	0	2 (40)	2 (18)		0.486	0.515	0.999
Other radiation	11 (42)	3 (100)	3 (60)	5 (45)		0.622	0.999	0.670
**Prior Treatment**^a^								
Anthracyclines	2 (7)	0	2 (22)	0		0.486	0.999	0.190
VEGF inhibitors	7 (27)	4 (67)	3 (33)	0		0.315	**0.0063**	0.074
Other cancer treatment	14 (54)	2 (33)	4 (45)	8 (73)		0.999	0.162	0.362
**ICI Therapy**^a^								
Nivolumab (anti-PD-1)	8 (31)	3 (50)	2 (22)	3 (27)		0.329	0.600	0.999
Ipilimumab (anti-CTLA-4)	1 (4)	0	1 (11)	0		0.999	0.999	0.450
Pembrolizumab (anti-PD-1)	8 (31)	3 (50)	2 (22)	3 (27)		0.329	0.600	0.999
Atezolizumab (anti-PD-L1)	2 (7)	0	1 (11)	1 (9)		0.999	0.999	0.999
Combination therapy (Nivolumab + Ipilimumab)	7 (27)	0	3 (33)	4 (36)		0.229	0.237	0.999
**Total ICI Cycles**^a^	9 ± 12	23 ± 17	4 ± 3	5 ± 4	**0.0011**	**0.0223**	**0.0222**	0.999
**Cycle 1 to clinical diagnosis (mo)**^a^	9 ± 13	22 ± 14	8 ± 13	3 ± 3	**0.0085**	0.069	**0.0071**	0.997
**Cycle 1 to death (mo)**^b^	16 ± 17	32 ± 20	6 ± 4	12 ± 12	**0.0078**	**0.0085**	**0.0392**	0.999
**Clinical diagnosis to death (mo)**^b^	7 ± 10	10 ± 8	3 ± 3	9 ± 12	0.325			
**Deceased**^a^	22 (85)	6 (100)	7 (78)	9 (82)		0.486	0.515	0.999

Values are expressed as mean ± standard deviation or *n* (%). Fisher’s exact test was used when comparing categorical variables and Student’s t-test or analysis of variance when comparing continuous variables. *P* values < 0.05 were considered significant. The *n* are defined as the following for *All*, *Possible*, *Probable*, and *Definite,* respectively: ^a^*n* = 26, 6, 9, 11; ^b^*n* = 22, 6, 7, 9. *Possible*, *Probable*, and *Definite* are based on the proposed reclassified myocarditis patient cohorts

*CTLA-4* Cytotoxic T lymphocyte-associated protein 4, *ICI* Immune checkpoint inhibitors, *mo* months, *PD-1* Programmed cell death protein 1, *PD-L1* Programmed cell death ligand-1, *VEGF*  Vascular endothelial growth factor

**Fig. 4:**
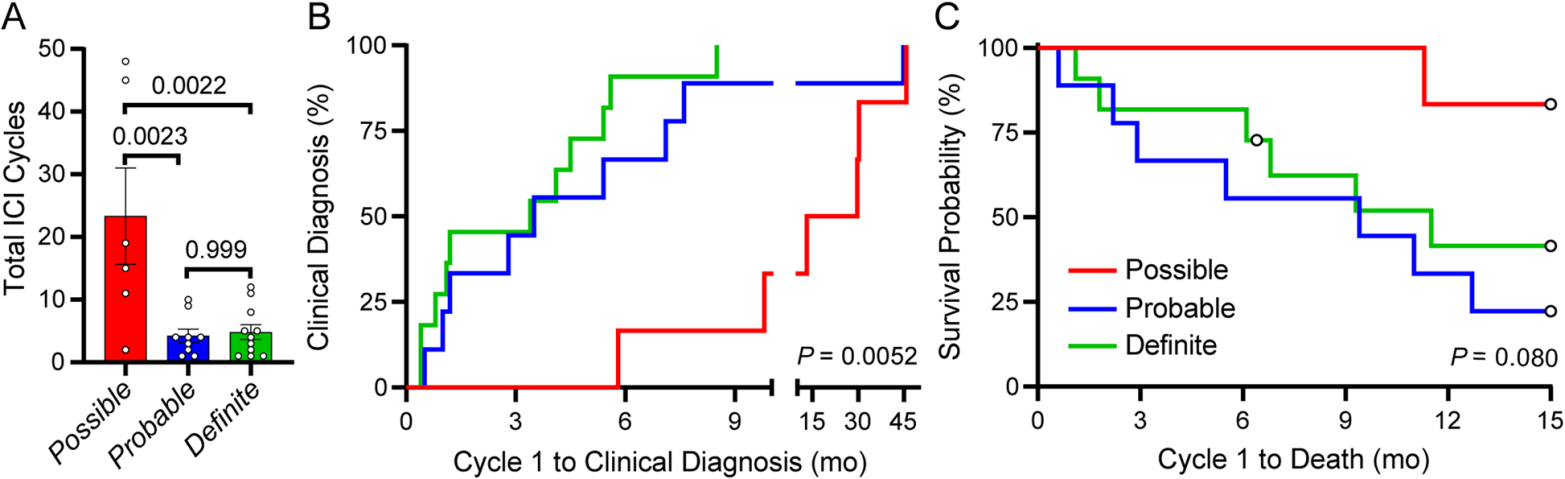
Total immune checkpoint inhibitor (ICI) cycles, time interval, and survival probability between first exposure to clinical diagnosis and death in reclassified patients. **A** Comparison of total ICI cycles. **B** Percent of patients with clinical diagnosis for myocarditis following cycle 1 of ICI treatment. **C** Percent survival probability of patients at 15 months following cycle 1 of ICI treatment. The *n* are defined as *Possible* (*n* = 6), *Probable* (*n* = 9), and *Definite* (*n* = 11) using the reclassified myocarditis patient cohorts. Open circle symbols represent censored patients in *Possible* (*n* = 5), *Probable* (*n* = 2), and *Definite* (*n* = 5) cohorts. Data were compared using the Mantel-Cox log-rank test with *P* < 0.05 being considered statistically significant

Under the potential reclassification, the total number of ICI treatment cycles until the clinical diagnosis of myocarditis would be significantly different among the three cohorts (*P* = 0.001). Post-hoc analysis revealed that patients reclassified into the *Probable* (*P* = 0.002) and *Definite* (*P* = 0.002) groups may present at earlier time points compared to the *Possible* group (Fig. 4A). In evaluating the number of months between cycle 1 to clinical diagnosis of myocarditis, there was a significant difference among the three groups (*P* = 0.005) with 2 group analysis showing that patients reclassified into the *Probable* (*P* = 0.047) and *Definite* (*P* = 0.003) groups may present after fewer cycles compared to the *Possible* group (Fig. 4B). For all 26 patients including those with potential reclassification, the time from cycle 1 of ICI treatment to clinical diagnosis occurred within 9 months for 100% of patients in both the *Probable* and *Definite* groups compared to only 25% for the *Possible* group. There was a trend (*P* = 0.08) in survival probability at 15 months among the three groups from the time following cycle 1 of ICI treatment that suggests potential reduced survival in the *Probable* and *Definite* groups (Fig. 4C). Almost 41% of patients died within 8 weeks of initial diagnosis despite steroid treatment (Table 4).

**Table 4:** Steroid treatment before and after potential reclassification

	**Initial Clinical Diagnosis**			**Potential Reclassified Diagnosis**		
**Steroid**	***Possible* (*n* = 8)**	***Probable*** **(*n* = 13)**	***Definite*** **(*n* = 5)**	***Possible*** **(*n* = 6)**	***Probable*** **(*n* = 9)**	***Definite*** **(*n* = 11)**
High	3 (38)	8 (62)	3 (60)	2 (33)	5 (56)	7 (64)
Other	1 (12)	3 (23)	1 (20)	1 (17)	3 (33)	1 (9)
None	4 (50)	2 (15)	1 (20)	3 (50)	1 (11)	3 (27)

Values are expressed as *n* (%). High dose steroids are defined as the equivalent of intravenous methylprednisolone of 1 gm or higher and Other steroids are defined as less than 1 gm

## Discussion

**Fig. 5:**
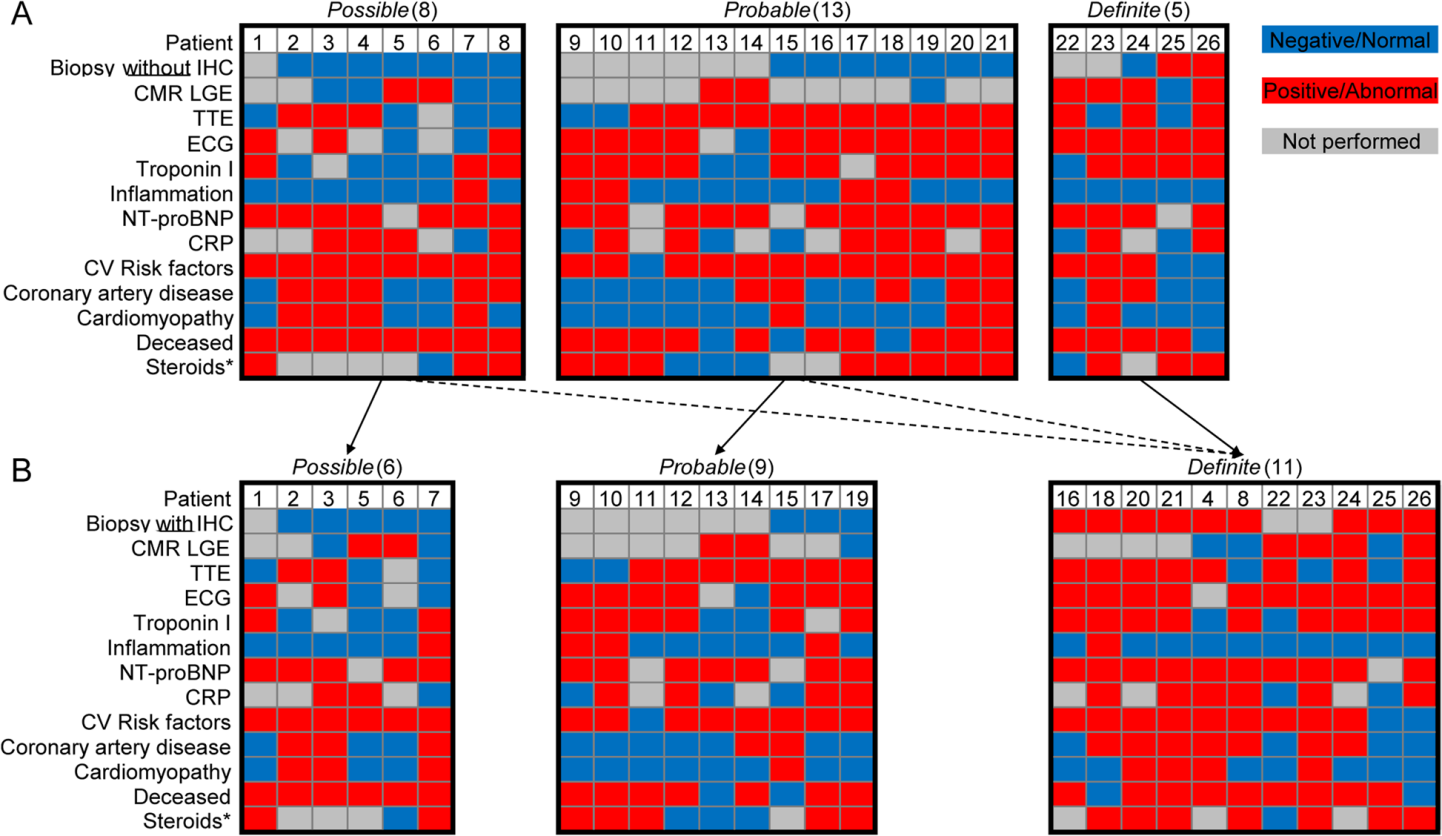
Myocarditis classification with and without supplemental immunohistochemistry (IHC) staining. Heatmaps of standard myocarditis classification criteria and selected variables from patients with immune checkpoint inhibitor-associated myocarditis. **A** Myocarditis classification without IHC and **B** potential reclassification with CD68 positive IHC. Each column represents characteristics from one patient (*n* = 26). Blue boxes represent negative/normal data, red boxes represent positive/abnormal data, and gray boxes represent studies not performed. *For steroids, red boxes represent high dose steroids that are defined as the equivalent of intravenous methylprednisolone of 1 gm or higher, blue boxes represent other steroids which are defined as a less than 1 gm, and gray boxes represent no steroids given. CMR = cardiac magnetic resonance imaging; CRP = C-reactive protein; CV = cardiovascular; ECG = electrocardiogram; LGE = late gadolinium enhancement; NT-proBNP = N-terminal pro-brain natriuretic peptide

While the Dallas criteria can identify a subset of myocarditis patients, this study demonstrates that quantification of macrophage abundance adds additional information that correlates with clinical presentation and assists in establishing the diagnosis of the ICI-associated myocarditis. In this study, we present 26 cases of suspected ICI-associated myocarditis with detailed clinical evaluation and outcomes. After applying standard accepted tools for the diagnosis and classification of ICI-associated myocarditis [14, 15], only 5/26 patients met criteria for *Definite* myocarditis. Of the 17 patients that underwent EMB, only 2 patients satisfied the Dallas criteria for myocarditis. When supplemental IHC staining in EMBs was performed, 7 additional patients could meet criteria for pathologic cardiac inflammation given positive CD68-IHC staining (> 50 CD68^+^ cells/HPF) and may be reclassified as having a *Definitive* diagnosis of ICI-associated myocarditis. The remaining 8 patients would maintain their original classification (Fig. 5A, B). These data highlight that deeper immunophenotyping of myocardial inflammation may increase the diagnostic yield of EMB.

Pathologic presentations of ICI-associated myocarditis may vary based on severity of disease, sampling bias, prior immunosuppressive treatment, cardiovascular co-morbidities, and concomitant cardiotoxic cancer therapies [21, 22, 27, 28]. The utility of EMB for the diagnosis of ICI-associated myocarditis has many potential limitations which are only partially explained by sampling bias and inter-observer variability. There are established limitations of the Dallas criteria [16], and likely contribute to the underdiagnosis of ICI-associated myocarditis. Other important clinical observations limiting EMB as a crucial diagnostic test in ICI-associated myocarditis include the perceived additional risks related to the procedure, availability of procedural expertise, and availability of pathology expertise to perform detailed IHC testing. Despite these limitations, major professional groups advising about cardiac safety during ICI therapy have recommended EMB as part of the diagnostic paradigm [9, 10, 14].

Although it has been shown that the diagnostic utility of the Dallas criteria can be improved by the use of IHC [17], this has not been well established for ICI-associated myocarditis. As a means to screen EMB for ICI-associated myocarditis, recent work by Palaskas et al. [28] proposes the use of light and electron microscopy in addition to IHC staining while Champion et al. [21] propose the use of the ISHLT (International Society of Heart and Lung Transplantation) criteria that is used for acute cellular rejection. Given the significant variability, it appears that ICI-associated myocarditis presents histologically along a spectrum. Hence, a gap in the literature is the lack of a “gold standard” for diagnosis, which presents uncertainty as to which classification scheme, H&E alone or H&E and IHC, is clinically superior.

Identifying the optimal IHC panel represents an important next step. However, there is no consensus for which IHC stains should be performed in cases of suspected ICI-associated myocarditis and several studies have reported a wide array of IHC staining including CD4, CD8, CD3, CD68, CD20, PD-1, and PD-L1 [21, 22, 27, 28]. While the heart is interspersed with a variety of immune cells in the steady state, the bulk of immune cells within the heart are comprised of tissue resident macrophages [18, 29]. Macrophage numbers in the heart are dynamic, increasing with aging, hemodynamic stress, and myocardial injury [30]. Myocardial infiltration of CD68^+^ macrophages has previously been described in ICI-associated myocarditis with rapid and late onset [27]. Notably, the proposed reclassified *Definite* patient cohort is associated with an earlier onset of myocarditis following cycle 1 of treatment compared to the *Possible* group. In addition, survival outcomes were close to achieving statistical significance with the *Definite* group trending towards lower survival probability compared to the *Possible* cohort. These findings serve to highlight that patients with *Definite* ICI-associated myocarditis may be at risk for earlier onset myocarditis and decreased survival probability. The seriousness of the potential clinical diagnosis reaffirms that early aggressive treatment should be instituted for patients with *Definite* ICI-associated myocarditis.

## Study limitations

This study requires additional validation of CD68 IHC staining in EMBs from patients with suspected ICI-associated myocarditis. Increased macrophage infiltration can be seen in other forms of cardiac injury such as acute myocardial infarction and heart failure. However, we anticipate that CD68 IHC staining will improve the sensitivity of diagnosing ICI-associated myocarditis in the appropriate clinical context. This limitation is not unique to macrophages since increased T-cell (CD3) and PD-L1 staining is also observed with non-myocarditis pathology [31]. Our study was limited by the small number of patients that underwent an EMB, although this is within clinical expectations as patients may already be presumed to have ICI-associated myocarditis or be too sick to undergo an EMB. We recognize that this is a retrospective study and clinical information is limited to what is recorded in the medial record and not all CMR studies had T1 and T2 mapping. Finally, we recognize inconsistencies between CMR and EMB biopsy findings that relate to sampling bias and inherent limitation of magnetic imaging without the use of T1 mapping and application of the modified Lake Louise Criteria [32, 33]. Our initial findings support the rationale of designing a prospective multi-center cohort study to address the limitations we have highlighted.

## Conclusions

Collectively, our findings highlight the possible utility of supplemental CD68 IHC staining when performing EMB to aid in the diagnosis of ICI-associated myocarditis. Given that ICI-associated myocarditis carries significant morbidity and mortality, early and accurate diagnosis is of paramount importance as it will improve the implementation of appropriate immunosuppressive therapy.

## Perspectives

### Competency in medical knowledge

Immune checkpoint inhibitor (ICI) myocarditis is associated with significant morbidity and mortality. Endomyocardial biopsy (EMB) is considered one of the gold standards for diagnosis; however, the sensitivity of biopsies and the pathological features that correlate with the clinical diagnosis of myocarditis remain incompletely defined. While the Dallas criteria can identify a subset of myocarditis patients, quantification of macrophage abundance adds additional information that correlates with clinical presentation and assists in establishing the diagnosis of the ICI-associated myocarditis.

### Translational outlook

Limitations exist with solely using Dallas criteria to diagnose ICI-associated myocarditis from EMB and these findings suggest that deeper immunophenotyping of myocardial inflammation may increase the diagnostic yield of EMB.

## Data Availability

All data generated or analyzed during this study are included in this published article.

## Abbreviations

ASCO: American Society of Clinical Oncology
CD68: Cluster of differentiation 68
CMR: Cardiac magnetic resonance imaging
CRP: C-reactive protein
CTLA-4: Cytotoxic T lymphocyte-associated protein 4
Dx: Diagnosis
ECG: Electrocardiogram
EMB: Endomyocardial biopsy
HPF: High powered field
ICI: Immune checkpoint inhibitors
ID: Identification
IHC: Immunohistochemistry
LVEF: Left ventricle ejection fraction
MRI: Magnetic resonance imaging
NCCN: National Comprehensive Cancer Network
NT-proBNP: N-terminal pro-brain natriuretic peptide
PD-1: Programmed cell death protein 1
PD-L1: Programmed cell death ligand-1
RAAS: Renin-angiotensin-aldosterone system
VEGF: Vascular endothelial growth factor
